# Supplementary material for: Heterogeneity of Cancer Stem Cells in Tumorigenesis, Metastasis, and Resistance to Antineoplastic Treatment of Head and Neck Tumours
Source: Cells. 2021 Nov 8;10(11):3068. doi: 10.3390/cells10113068 (PMC8619944; doi:10.3390/cells10113068)
Supplement: Supplementary file 1 [file cells-10-03068-s001.zip › cells-1343203-supplementary.pdf]

Supplementary Tables

Table S1. Main putative stem cell markers in Head and neck squamous cell carcinomas

**Marker**	**Gene Ontology**
CD44	Receptor for hyaluronic acid (HA). Mediates cell-cell and cell-matrix interactions through its affinity for HA, and possibly also through its affinity for other ligands such as osteopontin, collagens, and matrix metalloproteinases (MMPs). Adhesion with HA plays an important role in cell migration, tumor growth and progression.
MET	Hepatocyte growth factor receptor; Receptor tyrosine kinase that transduces signals from the extracellular matrix into the cytoplasm by binding to hepatocyte growth factor/HGF ligand. Regulates many physiological processes including proliferation, scattering, morphogenesis and survival.
SLC3A2	4F2 cell-surface antigen heavy chain; Required for the function of light chain amino-acid transporters. Involved in sodium-independent, high-affinity transport of large neutral amino acids such as phenylalanine, tyrosine, leucine, arginine and tryptophan.
Bmi1	Polycomb complex protein BMI-1; Component of a Polycomb group (PcG) multiprotein PRC1- like complex, a complex class required to maintain the transcriptionally repressive state of many genes, including Hox genes, throughout development.
Oct3/4	POU5F1 - POU domain, class 5, transcription factor 1; Transcription factor that binds to the octamer motif (5'-ATTTGCAT-3'). Forms a trimeric complex with SOX2 on DNA and controls the expression of a number of genes involved in embryonic development such as YES1, FGF4, UTF1 and ZFP206. Critical for early embryogenesis and for embryonic stem cell pluripotency
Nanog	Transcription regulator involved in inner cell mass and embryonic stem (ES) cells proliferation and self-renewal. Imposes pluripotency on ES cells and prevents their differentiation towards extraembryonic endoderm and trophectoderm lineages
CD133	Prominin-1; May play a role in cell differentiation, proliferation and apoptosis. Binds cholesterol in cholesterol- containing plasma membrane microdomains and may play a role in the organization of the apical plasma membrane in epithelial cells. Involved in regulation of MAPK and Akt signaling pathways.
ALDH1	Retinal dehydrogenase 1; Can convert/oxidize retinaldehyde to retinoic acid. Binds free retinal and cellular retinol-binding protein-bound retinal. May have a broader specificity and oxidize other aldehydes in vivo
CD90	THY1 - Thy-1 membrane glycoprotein; May play a role in cell-cell or cell-ligand interactions during synaptogenesis and other events in the brain
Sox2	Transcription factor that forms a trimeric complex with OCT4 on DNA and controls the expression of a number of genes involved in embryonic development such as YES1, FGF4, UTF1 and ZFP206. Critical for early embryogenesis and for embryonic stem cell pluripotency.
Podoplanin	Mediates effects on cell migration and adhesion through its different partners. During development plays a role in blood and lymphatic vessels separation by binding CLEC1B, triggering CLEC1B activation in platelets and leading to platelet activation and/or aggregation

Table S2. Expression of stemness genes and proteins in Head and Neck Cancer Stem Cells

Reference	Author(s)	Type of study	Study population	Experimental findings
11	Chen et al. 2009	in vitro and in vivo SCID mouse model	Biopsies of patients with HNSCC	Stemness genes (Oct-4, Nanog, Sox2, Klf4, Bmi-1 and Nestin) were upregulated in both ALDH1+ and CD44+/CD24-/ALDH1+ cells
26	Lim et al. 2012	in vitro and in vivo BALB/c nude mouse model	Biopsies from patients with HNSCC	A population of cells with a CSC-like phenotype isolated from HNSCC had upregulation of stemness markers OCT4 SOX2, Nestin and CK 5
43	Ravindran et al. 2015	in vitro and cross-sectional	Biopsies of 60 patients with oral squamous cell carcinoma<br><br>Cell lines: H314 and CAL27	Significant correlation was found between β-catenin expression and stage ($p < .01$), lymph node metastasis ($p < .004$) and histological grade ($p < .04$)<br><br>Oct-4 expression was significantly correlated with stage ($p < .01$) and tumor differentiation ($p < .001$)<br><br>Oct-4 staining was significantly higher in lymph node metastasis positive cases negative cases ($p <.009$)<br><br>Nanog expression was significantly higher in stage III to IV compared with stage I to II ($p < 0.009$)<br><br>Nanog expression was significantly correlated with lymph node metastasis ($p < .004$) and associated with histological grade ($p <.001$)<br><br>Involucrin expression was not found in carcinoma tissues showing intracellular expression of β-catenin
38	Mohanta et al. 2017	in vitro	Biopsies of patients with OSCC<br><br>Cell lines: UPCI:SCC029 B and AW13516, UPCI:SCC040	Cell lines enriched for expression of CD44 and CD147 cells has significantly elevated levels of Oct3/4 ($p < 0.001$)

			and CAL-27, and UPCI: SCC103 and UPCI: SCC16	
45	Koukourakis et al. 2012	in Vitro and cross-sectional	Biopsies from from 74 patients with HNSCC	N-Stage and Oct4 expression were significantly linked with presence of distant metastasis
27	Seino et al 2016	in vitro	Cell lines: OM-1, HOC621, and ZA	mRNA expression of the stem cell markers Sox2, Oct4 and Nanog was significantly highly expressed in CD44high cells (p<0.05, p<0.01, p<0.01)<br><br>mRNA expressions of Sox2, Oct4 and Nanog were significantly up-regulated in the CD44high/ALDH1high cells (p<0.01, p<0.001, p<0.01)<br><br>GSK3b is necessary for activity of CD44high/ ALDH1high cells:<br>● level of GSK3b was higher in the CD44high than the CD44low fraction<br>● siRNA knockdown of GSK3b inhibited both ALDH1 mRNA expression and enzymatic activity in CD44high/ALDH1high cells<br>● the number of tumor spheres was significantly reduced in GSK3b knockdown CD44high/ALDH1high cells (p<0.001)<br>● mRNA expressions for the stem cell markers Sox2, Oct4 and Nanog were significantly inhibited by siRNA knockdown of GSK3b in CD44high/ALDH1high cells
22	Lim et al. 2014	in vitro and in vivo mouse model	Cell lines: K3, K4, and K5	Knockdown of c-Met attenuated HNSCC stem-like cells traits<br>● Significant increase in c-Met expression was observed in ALDHhigh cells compared to ALDHlow cells<br>● Significantly more tumorspheres were formed from the Met high cells

				- cMet high cells also displayed enhanced transcriptional levels of stem cell markers (Oct4 and Sox2) - c-Met knockdown decreased the sphere-forming capacity of HNSCC stem-like cells and expression of stem cell markers (CD44, ALDH1, Oct 4 and Sox2) <br>Knockdown of c-Met enhanced cisplatin chemosensitivity of HNCSCs: - c-Met knockdown significantly decreased the Side Population cells in HNCSCs - c-Met knockdown decreased the expression of ABCG2 transporter gene <br>Knockdown of c-Met exerted antitumor effects in the xenograft mouse model of HNCSCs: - c-Met knockdown HNCSCs did not form tumors in the tongues of all of the injected mice - a significant decrease in the expression of CD44 and ALDH1-positive cells due to c-Met knockdown
30	Chen et al. 2011	in vitro	Cell lines: UD-SCC 1, UM-SCC -11B, UT-SCC -9, UT-SCC-22, UT-SCC-24A	Increased expression of stemness-related genes in Spheroid Derived Cells (SDC): mRNA levels of Oct3/4, Sox2 and Nanog were all significantly increased in SDC
21	Sun and Wang 2011	in vitro and in vivo NOD/SCID mouse model	Biopsies of patients with HNSCC	Self-renewal pathways were upregulated in c-Met1 HNSCC cells: BMI-1 expression was upregulated 6.9-fold in bulk HNSCC cells, 3.1-fold in c-Met-HNSCC cells, and 33.4-fold in c-Met+ HNSCC cells compared to normal oral epithelial cells
37	Michifuri et al. 2012	in vitro and cross-sectional study	Biopsies from 80 patients with OSCC	Sox2-positive staining was observed in all cases of OSCC<br><br>A Sox2 diffuse staining pattern was correlated with presence of lymph node metastasis ($P < 0.001$) and high histological grade ($P < 0.001$)

20	Bourguignon et al. 2012	in vitro	Biopsies from patients with HNSCC<br><br>Cell line: HSC-3	Detection of stem cell marker expression in tumorigenic CD44v3high/ALDH1high cells and in HNSCC Tumors:<br>● gene expression of Oct4, Sox2 and Nanog was significantly increased in CD44v3high/ALDH1high cells as compared with CD44v3low/ALDH1low cells or unsorted cells<br>● the expression of CD44v3, Oct4, Sox2 and Nanog expression was associated with advanced human HNSCC samples but not correlated with tumor grade or differentiation<br><br>There was a strong correlation between overexpression of CD44v3-Oct4-Sox2-Nanog and head and neck cancer progression:<br>● 50 of 66 advanced OSCC samples showed CD44v3 overexpression<br>● 40 of 66 showed Oct4 overexpression<br>● 38 of 66 showed Sox2 overexpression<br>● 45 of 66 showed Nanog overexpression<br>● These immunostaining results were statistically significant with a $p<0.02$ (for CD44v3 staining), $p< 0.01$ (for Oct4 staining), $p<0.02$ (for Sox2 staining), and $p <0.001$ (for Nanog staining) compared with CD44v3-Oct4-Sox2-Nanog detected in early (T1-T2) primary tumors ($n =44$) from HNSCC patient samples<br><br>Hyaluronan (HA)-induced Oct4-Sox2-Nanog complex formation in tumorigenic CD44v3high/ALDH1high cells:<br>● Immunoprecipitation results show a physical association between CD44v3 and Oct4-Sox2-Nanog<br>● treatment of CD44v3high/ALDH1high cells with HA promoted a complex formation between CD44v3 and all three stem cell markers<br>● Staining intensity of Nanog-Oct4-Sox2 in HA-treated cells was always higher than the control or anti-CD44 antibody-treated samples

				15–20-min HA treatment of CD44v3high/ALDH1high cells stimulated a significant amount of Oct4-Sox2-Nanog accumulation and complex formation in the nucleus - pretreatment with anti-CD44 antibody reduced HA-mediated Nanog-Oct4-Sox2 association with CD44v3 suggesting that Oct4-Sox2-Nanog membrane localization with CD44v3 induced by HA in CD44v3high/ALDH1high cells was CD44-dependent - HA-induced Nanog phosphorylation may be responsible for recruiting Oct4 and Sox2 complex formation and nuclear translocation <br>Role of Oct4-Sox2-Nanog in the regulation of miR-302 expression in HA-activated CD44v3high/ALDH1high cells: - The expression levels of miR-302a and miR-302b were strongly elevated in all 10 CD44v3high/ALDH1high cell-induced tumors - Oct4-Sox2-Nanog complex binds directly (or forms as part of a complex) to the promoter region of miR- 302 clusters in CD44v3high/ALDH1high cells following HA-CD44v3 activation - the level of both miR-302a and miR-302b was increased in CD44v3high/ALDH1high cells treated with scrambled sequence siRNA compared with those cells without HA treatment - HA-induced miR-302a/miR-302b expression was reduced when Oct4-Sox2-Nanog was downregulated - HA-CD44-activated Oct4-Sox2-Nanog signaling plays an important role in the production of miR-302a and miR-302b in CD44v3high/ALDH1high cells <br>Role of miR-302a/miR-302b in the expression of epigenetic regulators and survival proteins in tumorigenic CD44v3high/ALDH1high cells: - miR-302a and miR-302b were functionally

				coupled with the inhibition of AOF1/AOF2 and DNMT1 expression<br>● A basal level of AOF1/AOF2 and DNMT1 expression was detected in CD44v3high/ALDH1high cells transfected with negative control miRNA but not treated with HA or pretreated with anti-CD44 antibody followed by HA addition. Under these conditions, incomplete cutting of DNA by HpaII enzyme resulted in reduced/smaller DNA fragment formation suggesting the presence of a lower level of DNA global demethylation<br>● down-regulation of miR-302a or miR- 302b promoted both AOF1/AOF2 and DNMT1 up-regulation which then lead to a decrease in DNA global demethylation<br><br>Involvement of HA-CD44-regulated miR-302a/miR-302b in CSC-like properties in tumorigenic CD44v3high/ALDH1high Cells:<br>● HA-treated CD44v3high/ALDH1high cells formed a large number of spheres, ranging from 50 to 100 cells per spheroid<br>● In contrast, a much reduced number of spheres were detected in those cells treated with anti-CD44 antibody plus HA or no HA<br>● CD44v3high/ALDH1high cells that were dissociated from spheres were capable of growing and self-renewing after multiple passages from spheres<br>● The ability of cell growth and self-renewal appeared to be greatly enhanced in CD44v3high/ALDH1high cells treated with HA
12	Chen et al. 2010	in vivo (SCID mice and/or nude mice (BALB/ c strain) mouse	Biopsies from seven patients with HNSCC	HNSCCALDH1+ cells displayed higher levels of Bmi-1 at both the mRNA and protein levels<br><br>ALDH1+ cells had increased expression of stemness genes:<br>● the expression levels of embryonic stem cell-specific genes, including Oct-4, Nanog, Sox-2,

		model		Klf-4 and Nestin, were higher in HNSCC ALDH1+ cells than those observed in ALDH1- cells ($p < 0.05$)<br><br>Bmi-1 knockdown reduced cancer stem cell properties and expression of stemness genes:<br>● foci formation, migration/invasion and sphere formation were significantly reduced in sh-Bmi-1-treated HNSCC ALDH1+ cells compared to sh-Luc-treated HNSCC ALDH1- cells ($p < 0.05$)<br>● Oct-4, Nanog, Sox2, Nestin, Mushashi, c-Myc and β-catenin and drug resistant genes (MDR-1, MRP-1 and ABCG2) were significantly down-regulated in HNSCC ALDH1+ cells treated with sh-Bmi-1 ($p < 0.05$)<br><br>Bmi-1 knockdown reduced radio-/chemo-resistance of ALDH1+ cells in vitro and in vivo:<br>● the chemotherapeutic effect of taxol was significantly stronger in HNSCC ALDH1+ cells treated by sh-Bmi-1 ($p < 0.05$) compared to control<br>● The IR effect on HNSCC ALDH1+ cells treated with sh-Bmi-1 was significantly stronger compared to either untreated HNSCC ALDH1+ cells or HNSCC ALDH1+ treated with a scrambled control ($p < 0.05$)<br>● the tumor volumes in the HNSCC ALDH1+-transplanted mice were significantly decreased in mice treated with both IR and sh-Bmi-1 compared to mice exposed to IR alone ($p < 0.05$)<br>● tumor volumes in the Bmi-1-shRNA-treated HNSCC ALDH1+ innoculated mice who received IR treatment were significantly lower than those of untreated HNSCC ALDH1+-transplanted mice, HNSCC ALDH1+ transplanted mice treated with IR alone, or HNSCC ALDH1+ transplanted mice treated with the control vector and IR ($p < 0.05$)
7	Yanamoto	in vitro	Cell line:	The expression of Oct-4 and EpCAM was significantly

	et al. 2011		SCC25	higher in sphere forming SP cells than in parental and non-SP cells (p<0.01)
33	Shigeishi et al. 2013	in vitro	Cell lines: CA1 and LUC4	GSK3b was necessary for the self renewal of CSCs:<br>● significantly greater numbers of tumor spheres were formed by EMT CSCs than holoclones of EPI CSCs<br>● The number of tumor spheres formed by EMT CSCs correlated with the size of the fraction of EMT CSCs present in the unsorted parental population (7.5%, 16.5%, and 21.0% in CA1, MET2, and LUC4, respectively)<br>● In the high sphere-forming MET2 and LUC4 lines, the CD44high/ESAlow cells had low levels of phosphorylated GSK3b whereas in the low sphere-forming CA1 line the CD44high/ESAlow cells exhibited higher expression of phosphorylated GSK3b<br>● EMT cells had significantly higher levels of CD44 and lower levels of phosphorylated GSK3b<br>● inactivation of GSK3b decreased tumor sphere formation by CD44high/ESAlow cells and the number of holoclones formed by CD44high/ESAhigh cells<br>● inactivation of GSK3b significantly reduced Oct4, Sox2 and Nanog in CD44high/ESAlow and Oct4 and Nanog in CD44high/ESAhigh cells<br><br>Inactivation of GSK3b induced cell differentiation:<br>● The percentage of both CD44high/ESAlow and CD44high/ESAhigh cells decreased following GSK3b inactivation, but the percentage of CD44low cells consistently increased suggesting a shift of CSCs into differentiation<br>● GSK3b inactivation produced significantly increased levels of epithelial differentiation markers (Involucrin and Calgranulin B) in CD44high/ESAhigh cells

				CD44 and RHAMM are required for self renewal of CSCs and for the regulation of GSK3b:<br>● CD44high/ESAhigh cells showed higher expression of RHAMM than CD44high/ESAlow cells<br>● Both RHAMM and CD44 knockdown resulted in phosphorylation (inactivation) of GSK3b and phosphorylation (activation) of ERK1/2<br>● Holoclone formation by CD44high/ESAhigh cells was significantly inhibited by both RHAMM and CD44 knockdown<br>● Knockdown of CD44, but not knockdown of RHAMM, caused a significant decrease in the number of tumor spheres formed by CD44high/ESAlow cells<br>● CD44 and RHAMM knockdown significantly decreased expression of Sox2, Nanog and Oct4 in CD44high/ESAhigh cells and also, upregulated expression of the differentiation markers Involucrin and Calgranulin B
39	Tsai et al. 2014	in vitro and in vivo BALB/c nude mouse model	Biopsies from patients with OSCC<br><br>Cell lines: SAS, FaDu, OECM1, SCC4, HSC3, OC3 , Ca922, SCC25, and GNM<br><br>SG (normal gingiva cell line)	Oct4 upregulated in OSCC cell lines:<br>● Oct4 mRNA and protein were detectable in OSCC cell lines SSC4 and SAS OSCCs. It was lower or undetectable in normal oral epithelial cells<br><br>Oct4 overexpression enhanced cell proliferation, invasiveness and colony formation.<br><br>Oct4 overexpression enhanced in vivo tumorigenicity and mesenchymal traits in OSCCs:<br>● OSCC cell lines that over-expressed Oct4 showed a significant increase in tumorigenic capacity in vivo ($p<0.05$)<br>● There was reduced expression of E-cadherin, but enhanced expression N-cadherin and Slug in OSCC cells that over-expressed Oct4<br>● Down-regulation of Oct4 decreased the self renewal capacity, matrigel invasion and anchorage independent growth of OSCC-CSCs and significantly slowed down tumor growth

				in vivo (p<0.05)<br><br>Upregulation of Oct4 expression in recurrent and metastatic OSCC patients:<br>● Increased tumorigenicity compared to non-tumor samples from the same patient<br>● Was evident in metastatic lymph nodes when compared with primary tumors
35	Lim et al. 2012	in vitro and in vivo BALB/c nude mouse model<br><br>Cross-sectional study	Biopsies from 71 small (pathologically T1–2) SCCOT patients<br><br>Cell line: SNU1041	Constitutive activation of c-Met promoted the invasion of tongue cancer cells in vitro:<br>● Constitutive activation of c-Met significantly enhanced the migration and invasion (p<0.01) of SNU1041 cell<br>● The levels of MMP-1, -2, and -12 mRNAs increased in SNU1041-c-Met cells (p<0.01)<br>● Constitutive activation of c-Met promoted the growth of tongue carcinoma cells in vitro and in vivo (P<0.01)
24	Chiou et al. 2008	in vitro and in vivo BALB/c nude mouse model<br><br>Cross-sectional study	Bopsies from patients with OSCC<br><br>Cell lines: SAS and OECM1	Elevated expression of progenitor/stem cell genes and markers in oral cancer stem-like cells (OC-SLC)<br>● The amounts of Oct-4, Nanog and Nestin transcripts in enriched OC-SLC were significantly increased compared with that of the parental OSCC cells<br>● OC-SLC cells were positively stained with CD133 and CD117<br>● Most of the enriched OC-SLC from SAS and OECM1 were ABCG2 positive<br><br>Increased Oct-4 expression was positively correlated with advanced stages and medium/poor differentiation of primary OSCC tumors but not lymph node metastases.<br><br>Increased nuclear staining of Oct-4 was observed in grades III and IV OSCC compared to grades I and II tumors<br><br>Elevated expression of Nanog and CD133 were positively associated with high-grade OSCC and medium/poorly differentiated tumors (all p<0.05)
41	Chikamats	in vitro	cell lines: Gun-	CD44+ cells constituted 2.6% of the parental Gun-1

	u et al. 2012		1 and Ca9-22	cell line and 8.5% of the parental Ca9-22 cell line<br><br>CD44+ cells showed increased expression levels of stemness genes (Nanog, Bmi-1, Notch-1 and Oct-4) compared with CD44- cells in both Gun-1 and Ca9-22
8	Zhang et al. 2009	in vitro and in vivo BALB/C nude mouse model	Biopsies from patients with OSCC<br><br>Cell lines: Tca8113, Tca/cisplatin, NT, NTCR, NB, NBCR, TSCC, TL, and Tb	Differential gene expression between SP and non-SP cells:<br>● Expression of CK19, Bmi-1, NSPC1, Oct-4 and CD44 were significantly higher in SP cells than in non-SP cells, whereas the expression of INVO and CK13 was significantly lower in the SP cells compared to non-SP cells
44	Lee et al. 2014	in vitro and in vivo immunodeficient mouse model	Biopsies from patients with HNSCC<br><br>Cell lines: SNU1041 and FaDu	SOX2 promoted proliferation of HNSCC cells via cyclin B1 upregulation:<br>● overexpression of Sox2 increased the growth rate of HNSCC cells in vitro<br>● overexpression of Sox2 increased the transcriptional and translational level of cyclin B1 in HNSCC<br>● the enhanced proliferation of cells by Sox2 was reversed by transient suppression of cyclin B1<br><br>Sox2 enhanced stem cell traits of HNSCC cells in vitro:<br>● Oct4 and Nanog were positively regulated by Sox2<br>● Sox2 overexpression caused SNU1041 cells to show accelerated tumor sphere formation<br>● Sox2-positive tumor cells co-expressed the stemness marker CD44<br><br>Sox2 was highly expressed in HNCSCs and enhanced self renewal and chemoresistance:<br>● Sox2 expression in ALDHhigh cells was significantly higher than in ALDHlow cells<br>● Sox2 downregulation in HNCSCs was

				associated with downregulation of Oct4 and Nanog,<br>● sphere-forming capacity was significantly reduced in HNCSC with knockdown of Sox2<br>● Sox2 increased the number of cisplatin-resistant tumor cells<br>● cell viability after cisplatin treatment was decreased by Sox2 downregulation<br>● ABCG2 was weakly expressed in Sox2-deficient CSCs compared with control CSCs<br><br>Sox2 increased the invasiveness of HNCSCs through epithelial-mesenchymal modulation:<br>● Sox2 downregulation induced E-cadherin expression and decreased vimentin expression<br>● SNAIL was downregulated when Sox2 was knocked down in HNCSCs and resulted in decreased invasiveness in the Transwell assay<br><br>Knockdown of Sox2 inhibited xenografted tumor growth in vivo:<br>● xenografted HNCSC shSox2 cells exhibited reduced tumorigenicity compared with control HNCSCs<br>● Sox2 knockdown decreased the proliferative ability and stemness properties in the xenografted tumor tissue
50	Reers et al. 2013	in vitro	Cell lines:<br>PCI-I,<br>BHYUT-SCC 12A/B,<br>UT-SCC 60A/B,<br>UT-SCC 74A/B	Enrichment of Oct-4 expressing cells after exposure to chemotherapeutic agents:<br>● The Oct-4 cell population of untreated PCI-I cells showed an average cell rate of 21.2% (SD 9.3, n = 11)<br>● Exposure of PCI-I cells to 200 lM 5-fluorouracil for 48 h increased the mean number of Oct-4 positive cells (21.2% to 33.8%; SD 5.5, P = 0.005), whereas the mean number of vital cells was decreased to 62.0%<br>● In the cisplatin-treated cell fraction with a reduction to 30.4% in all PCI-I-cells, there was a slight increase of Oct-4 positive cells (mean: 36.5%; SD 9.0, P = 0.0044)<br>● In the paclitaxel-treated cell fraction, treatment

				with 10 lM Paclitaxel resulted in a reduction in cell rate, down to 18.7% in all cells, as well as a pronounced increase of Oct-4+-cells (mean: 57.9%; SD 13.0, P = 0.0011)

Experimental findings supporting the upregulation and expression of stemness genes and proteins and the possible biological pathways which maintain stemness characteristics in head and neck cancer stem cells.
HNSCC = head and neck squamous cell carcinoma, *OSCC = oral squamous cell carcinoma ,* ***SCC = squamous cell carcinoma,* **HNCSC = head and neck cancer stem cells, *SP = side population, *EMT = epithelial-mesenchymal transition*

Table S3. Activation and upregulation of the Wnt/β-catenin pathway in Head and Neck Cancer Stem Cells

Reference	Author(s)	Type of study	Study population	Experimental findings
26	Lim et al. 2012	in vitro and in vivo BALB/c nude mouse model	Biopsies from patients with HNSCC	Retinoic acid (RA) downregulated the Wnt/β-catenin signalling pathway:<br>● Administration of All-trans-retinoic-acid (ATRA) in HNCSCs decreased expression of β-catenin<br>● ATRA administration decreased the activity of a Wnt/β-catenin-dependent reporter gene<br><br>Downregulation of Wnt/β-catenin by RA reduced stemness properties:<br>● RA inhibited proliferation of HNCSCs and reduced sphere formation<br>● RA suppressed the expression of stem cell markers Oct4, Sox2, Nestin and CD44<br>● Cisplatin + RA increased HNCSC sensitivity to cisplatin compared to cisplatin or ATRA alone ($p<0.01$).
43	Koukourakis et al. 2012	in Vitro and cross-sectional	Biopsies from from 74 patients with HNSCC	Integrin-b1 expression was significantly linked with the presence of distant metastasis
46	Warrier et al. 2014	in vitro	Cell lines: Hep2 and KB	Inhibition of the Wnt pathway by the Wnt antagonist sFRP4 decreased stemness and chemoresistance in HNCSC:<br>● Addition of sFRP4 alone, or in combination with cisplatin, drastically disrupted the formation of secondary spheres and there was no clonogenic expansion<br>● Expression of the CSC-specific marker CD44 was reduced markedly in sFRP4-treated and in sFRP-Cis-treated Hep2 and KB spheroids<br>● sFRP4, cisplatin and combination treated Hep2 and KB spheroids demonstrated a reversal of EMT with the accumulation of E-cadherin and

				a reduction in N-cadherin levels<br>● The molecular markers Twist and Snail were downregulated in spheroids treated with sFRP4 and cisplatin<br>● The expression of the ABC genes (ABCG2 and ABCC4) were reduced in sFRP4-cisplatin-treated Hep2 spheroids<br>● The combination of cisplatin and sFRP4 effectively reduced cell viability to 25% in both Hep2 and KB spheroids. The caspase-3/7 assay showed induction of apoptosis by the activation of caspase 3/7 enzyme and was maximal in the sFRP4+Cisplatin treated cells
9	Song et al. 2010	in vitro and in vivo nude mouse model	Cell lines: 686LN, M3a2 and M4e	Upregulation of β-catenin:<br>● the activity of β-catenin-dependent transcription was significantly elevated in SP cells compared to non-SP cells
47	Lee et al. 2014	in vitro and in vivo immunodeficient mouse model	Cell lines: SNU-1041 and FaDu	Wnt/β-catenin signalling was differentially expressed in primary HNSCC tissues relative to normal tissue:<br>● Cytoplasmic/nuclear β-catenin was expressed in a subpopulation of tumor cells in HNSCC (8.1%) but in none of the normal head and neck tissues<br>● Cytoplasmic/nuclear β-catenin was localized to tumor cells expressing CD44 and ALDH1, suggesting that cytoplasmic/nuclear β-catenin was preferentially expressed in HNSCC cells with stem cell traits<br><br>Wnt/β-catenin signalling promoted proliferation of HNSCC cells:<br>● Overexpression of β-catenin resulted in accelerated growth rate and proliferation of HNSCC cells in culture<br><br>Ectopic expression of β-catenin generated stem-like HNSCC cells:<br>● β-Catenin-overexpressing HNSCC cells formed 7x more spheres<br>● Protein and mRNA levels of Oct4, Sox2 and

				CD44 were up-regulated in β-catenin-overexpressing HNSCC cells<br>● β-catenin-overexpressing HNSCC cells were significantly more resistant to cisplatin than control HNSCC cells.<br><br>Wnt/β-catenin signalling was activated in HNSCC stem-like cells:<br>● spheroid, but not adherent HNSCC stem-like cells, displayed nuclear immunostaining of β-catenin<br>● Inhibition of Wnt/β-catenin signalling with DKK1 resulted in decreased mRNA levels of nuclear β-catenin target genes such as cyclin D1 and c-Myc<br>● Wnt3A, a pharmacological activator of Wnt signalling, increased the sphere formation of HNCSCs<br><br>Disruption of β-catenin impaired HNCSCs:<br>● Knockdown of β-catenin in HNCSCs resulted in a significant reduction in sphere formation capacity<br>● reduced expression of various putative stem cell markers, including Oct4, Sox2 and CD44<br>● Knockdown of β-catenin significantly decreased chemoresistance<br><br>Targeting β-catenin suppressed the tumor growth of HNCSCs in vivo:<br>● HNCSCs with β-catenin shRNA displayed a marked decrease in tumor growth and tumorigenic potential<br>● palpable tumor masses developed in 50% (3 of 6) of mice injected with control vector-transduced HNCSCs whereas only 17% (1 of 6) of shβ-catenin HNCSCs formed tumors when 10^4 cells were injected<br><br>β-catenin directly activated Oct4 transcription in HNCSCs:<br>● Forced expression of Oct4 in shβ-catenin

				HNCSCs restored tumor sphere formation and rescued the impaired expression of stemness markers caused by β-catenin knockdown<br>● Tumor growth in vivo that was impaired by β-catenin downregulation was rescued by forced expression of Oct4<br>● Oct4 knockdown attenuated the expression of stemness markers (Sox2 and CD44), self-renewal capacity and cell proliferation

Experimental findings supporting the activation and upregulation of the wnt/β-catenin pathway in head and neck cancer stem cells.

**HNSCC = head and neck squamous cell carcinoma, *OSCC = oral squamous cell carcinoma , *HNCSC=head and neck cancer stem cells, *SCC = squamous Cell Carcinoma, *SP = side population, *EMT = epithelial-mesenchymal transition*

Table S4. Epithelial Mesenchymal Transition in Head and Neck Cancer Stem Cells

Reference	Author(s)	Type of study	Study population	Experimental findings
11	Chen et al. 2009	in vitro and in vivo SCID mouse model	Biopsies of patients with HNSCC	EMT pathway and EMT-related genes (especially Snail) were significantly activated in HNSCC ALDH1+ cells ($p<0.05$)<br><br>CD44+/CD24+/ALDH1+ cells exhibited a shift to EMT phenotypes with downregulation of E-cadherin and upregulation vimentin and Snail
38	Mohanta et al. 2017	in vitro	Biopsies of patients with OSCC<br><br>Cell lines: UPCI:SCC029 B and AW13516, UPCI:SCC040 and CAL-27, and UPCI: SCC103 and UPCI: SCC16	There was a significantly higher expression of EMT markers (Vimentin, Snail, and COL3A1; $p \leq 0.001$) in poorly differentiated SCC (PDSCC) and in moderately differentiated SCC (MDSCC) compared to well differentiated SCC (WDSCC)<br><br>There was a significantly elevated gene expression of N-cadherin, MMP2 and MMP9 ($p \leq 0.001$) in PDSCC than MDSCC and WDSCC
17	Biddle et al. 2011	in vitro and in vivo NOD/SCID mouse model	Biopsies from patients with OSCC<br><br>Cell line: CA1 (from OSCC of the floor of mouth)	OSCC cells plated at low density in adherent cultures formed a range of colony morphologies:<br>● CD44high/ESAhigh cells grew as self-renewing holoclones and the CD44low cells formed small abortive/differentiating paraclone colonies<br>● Individual elongated fibroblast cells lying outside the compact holoclone colonies were identified and were found to have a CD44high/ESAlow staining pattern<br>● The cells around the colony peripheries formed spheres whereas those in the center of the colonies did not form spheres, thereby confirming the presence of CD44high/ESAlow EMT CSCs at the colony peripheries<br>● Elongated cells at the colony peripheries, but not cell in the centre of the colonies, expressed Vimentin.

				An EMT–related gene expression pattern in the CD44high/ESAlow cells:<br>● CD44high/ESAlow cells formed 10x more spheres than the CD44high/ESAhigh cells, and 80x more than the CD44low cells<br>● CD44high/ESAlow cells showed higher rates of migration in 3-dimensional in vitro transwell migration assays<br>● CD44high/ESAlow cells had greater expression of EMT markers (Vimentin, Twist, Snail and Axl), and lower expression of the epithelial-specific genes (E-cadherin, Calgranulin B, Involucrin, and Keratin 15)<br><br>CSCs with an EMT phenotype were present in cell populations freshly isolated from OSCC:<br>● Cells explanted from each of three tumors were found to contain a CD44high/ESAlow cell populations that represented 28.0%, 8.2% and 5.1% of the total population<br>● The CD44high/ESAlow cells of all three tumors showed greater expression of Vimentin and Twist and less expression of E-cadherin<br><br>The two CSC phenotypes exhibited distinct in vivo behaviors that reflected their in vitro properties:<br>● Both phenotypes exhibited tumour initiating ability in vivo but only the EMT phenotype showed lymph node infiltration.<br>● Analysis of the tumors showed a return to a heterogeneous cell population indicating each CSC phenotype could repopulate the other in vivo<br><br>There was switching between the two CSC phenotypes:<br>● 100% of CD44high/ESAhigh clones were bipotent and able to produce both non-EMT and EMT cell populations<br>● Only a fraction of the CD44high/ESAlow clones (50% in CA1 and 29% in Met1) were able to produce mixed populations suggesting

				that those EMT CSCs which are capable of undergoing MET lie at the ESAhigh end of the CD44high/ESAlow cell population<br>● CD44high/ESAlow bipotent EMT CSCs were ALDH1+
16	Lee et al. 2017	in vitro and in vivo (B10;B6-Rag2−/−Il2rg−/−) mouse model	Biopsies of patients with HPV-negative OSCC	Higher mRNA levels of EMT regulators (TWIST1 and Bmi-1) and the EMT-inducing genes (ZEB1, ZEB2, SNAIL1 and SNAIL2) were observed in CD44+ cells compared to CD44− cells
27	Seino et al 2016	in vitro	Cell lines: OM-1, HOC621, and ZA	CD44high/ALDH1high cells exhibited marked Vimentin expression and mesenchymal features:<br>● mRNA expression of Vimentin was significantly increased in the CD44high/ALDH1high as compared to the CD44high/ALDH1low cells, while the level of expression of E-cadherin was significantly decreased in CD44high/ALDH1high cells ($p<0.01$)<br>● the mRNA expression of Snail was significantly up-regulated in CD44high/ALDH1high cells ($p<0.05$)<br>● spindle-shaped cells that were positive for vimentin and negative for E-cadherin were observed among the CD44high/ALDH1high cells
30	Chen et al. 2011	in vitro	Cell lines: UD-SCC 1, UM-SCC -11B, UT-SCC -9, UT-SCC-22, UT-SCC-24A	Spheroid-derived cells (SDC) acquired features of myofibroblasts and may have undergone EMT:<br>● Cell lines with high ALDH1 expression had a relatively high frequency of Vimentin- and α-SMA-expressing cells which suggests that these cell lines had mesenchymal characteristics<br>● All SDC contained more α-SMA and Vimentin positive cells than the corresponding parental cell lines<br>● SDC showed significantly increased proportions of low or negative E-Cadherin expressing cells compared to their monolayer

				derived counterparts<br>● Snail1 was significantly increased in all SDC generated from the five different HNSCC cell lines
33	Shigeishi et al. 2013	in vitro	Cell lines: CA1 and LUC4	CD44high/ESAhigh (5%) and CD44high/ESAlow (21%) phenotypes were present in SCC cell lines:<br>● Each of the cell lines contained cells with high expression of CD44 that were either:<br>(a) ESAlow and exhibited a spindle-like appearance, had high expression of Snail, Vimentin and Axl and low expression of E-cadherin (designated as EMT CSC)<br>(b) ESAhigh cells formed holoclones, had high expression of E-cadherin, low expression of Snail and Vimentin, and grew faster in adherent culture conditions than CD44high/ ESAlow cells (designated as EPI CSC)

Experimental findings supporting the ability of head and neck cancer stem cells in acquisition of a migratory phenotype and enhance metastatic potential by undergoing Epithelial Mesenchymal Transition.

**HNSCC = head and neck squamous cell carcinoma, *OSCC = oral squamous cell carcinoma , *HNCSC=head and neck cancer stem cells, *SCC = squamous Cell Carcinoma, *SP = side population, *EMT = epithelial-mesenchymal transition*

Supplementary Table S5
Influence of Endothelial cell derived growth factors on Head and Neck Cancer Stem Cells

Reference	Author	Type of study	Study population	Experimental findings
48	Xu et. al 2017	In vitro and in vivo (NCr-nu/ nu) mouse model	Cell lines: UM-SCC-1, SCC-116, CAL27 and FaDu	EGF promoted EMT:<br>● SCC-1 and SCC-116 underwent typical mesenchymal-like morphological changes characteristic of the EMT phenotype in response to EGF stimulation<br>● EGF-induced EMT in SCC-1 cells was confirmed by a dose-dependent decrease in the expression of E-cadherin with a simultaneous increase in the expression of Vimentin<br>● EGF-induced EMT was characterized by increased expression of the EMT regulatory transcription factors (Zeb1 and Slug) and a decrease in the epithelial marker ZO-1<br><br>EGF promoted the acquisition of stem-cell like properties:<br>● EGF stimulation upregulated CD44 expression and significantly enriched the fraction of CD44+/CD24low/− cells<br>● EGF significantly enhanced the expression of aldehyde dehydrogenase 1 (ALDH1) and Bmi-1<br>● EGF-induced EMT correlated with increased expression of stem cell-related genes ALDH1 and Bmi-1<br><br>EGF-induced EMT and CSC-like cell properties depended on aerobic glycolysis:<br>● pretreatment of SCC-1 cells with 2-deoxy-glucose (2-DG), a glycolysis inhibitor, reduced both the basal and EGF-stimulated lactate production in a dose-dependent manner<br>● the presence of 2-DG significantly prevented mesenchymal-like cell morphological changes in EGF-stimulated SCC-1 cells ($p<0.05$)<br>● Down-regulation of E-cadherin and upregulation of Vimentin by EGF were reversed by 2DG treatment<br>● 2-DG treatment markedly reduced the

				percentage of CD44+/CD24low subpopulations<br><br>Blocking glycolysis that had been suppressed by EGF facilitated metastasis of OSCC cells to regional cervical lymph nodes in vivo:<br>● the presence of EGF enhanced the expression of ALDH1, Vimentin, and PDK1 in tumors of the tongue in situ and increased the incidence of cervical lymph node metastasis (70% versus 20% of the control group; p=0.038)<br>● metastatic tumor cells in the cervical lymph nodes positively expressed Vimentin, ALDH1 and PDK1<br>● 2-DG treatment had no obvious inhibitory effects on tumor growth in situ but reduced the degree and incidence of EGF-facilitated cervical lymph node metastases (30% versus 70%); the difference was not significant (p=0.101)
28	Krishnamurthy et al. 2010	n vitro and in vivo SCID mouse model	Cell lines: UM-SCC-1, UM-SCC-74A, UMSCC-74B, UM-SCC-17A, UM-SCC-17B, UM-SCC-11B	ALDH+ cells in HNSCC were seen in tumor islands in close proximity to blood vessels:<br>● the majority of the CSCs (approx. 80%) were found in the perivascular area<br><br>Endothelial cell-derived growth factor milieu promoted proliferation, survival and self-renewal of HNCSC:<br>● The proliferation of both ALDH+/CD44+ and ALDH−/CD44− cells cultured in low attachment conditions was enhanced by exposure to endothelial cell conditioned medium<br>● Endothelial-cell secreted factors enhanced expression of Bmi-1 in ALDH+/CD44+ cells<br>● A 3-fold increase in the number of orospheres was observed in the group treated with endothelial cell conditioned medium ($P < 0.001$) compared to untreated controls<br>● Selective ablation of blood vessels reduced the proportion of cancer stem cells<br>● The fraction of ALDH+/CD44+/Lin− cells within xenograft tumors was significantly reduced when endothelial cells were selectively

				ablated
49	Zhang et al. 2014	in vitro and in vivo SCID mouse model	Cell lines: UM-SCC-1, UMSCC-11A, UM-SCC-11B, UM-SCC-14A, UM-SCC-14B, UM-SCC-17A, UM-SCC-17B, UM-SCC-22A, UM-SCC-22B, UM-SCC-74A and UM-SCC-74B	Endothelial cell-secreted EGF induced EMT of squamous cell carcinoma cells:<br>● Tumor cells approaching blood vessels exhibited features of EMT as characterized by low E-cadherin and high Vimentin expression<br>● Tumor cells within the blood vessel lumen reversed back to an epithelial phenotype characterized by high E-cadherin and low Vimentin, suggestive of MET<br>● Tumor cells in close proximity to blood vessels expressed the stem cell markers ALDH and CD44, a phenomenon enhanced when the tumor cells were within the lumen of the blood vessel<br>● EGF upregulated PEGFR expression<br>● P-Akt and P-ERK gene expression changes were consistent with EMT<br>● EGF decreased the expression of an epithelial marker (Desmoplakin), increased the expression of a mesenchymal marker (Vimentin) and activated expression of EMT transcriptional factors (Snail, Twist)<br><br>Endothelial cell-secreted EGF induced EMT through PI3K/Akt signaling:<br>● EGF and endothelial cell-secreted factors potently induced activation of STAT3, Akt and ERK<br>● Inhibition of PI3k/Akt signaling prevented EGF- and endothelial cell-induced Snail expression<br><br>Endothelial cell-secreted EGF endowed epithelial tumor cells with stem-like characteristics:<br>● EGF supplementation induced a significant increase in the number of orospheres when compared to controls ($p<0.05$)<br>● Recombinant human EGF, or the full milieu of growth factors secreted by endothelial cells, induced expression of Bmi-1<br>● EGF increased significantly the fraction of

				ALDH+/CD44+ cells ($p<0.05$)<br><br>Endothelial cell-secreted EGF enhanced tumor growth and the CSC fraction in vivo:<br>● EGF silencing in endothelial cells was sufficient to slow down tumor growth<br>● Tumors vascularized with EGF-silenced endothelial cells showed a significant reduction in the proportion of ALDH+ cells ($p<0.05$), were more differentiated and were less invasive
29	Campos et al. 2012	in vitro	Cell lines: UM-SCC-22A) and metastatic cell line (UM-SCC-22B	Endothelial cell secreted factors enhanced the survival of HNCSC:<br>● endothelial cell conditioned medium protected ALDH+/CD44+ cells ($P<0.05$) against anoikis that had been induced by a lack of anchorage or serum starvation<br>● VEGF secreted by endothelial cells enhanced the proliferation of primary, but not metastatic, HNCSC<br>● exposure of ALDH+/CD44+ cells to endothelial cell conditioned medium induced potent and fast phosphorylation of Akt<br>● exposure of control (ALDH−/CD44−) cells to the conditioned medium induced activation of Akt only in cells derived from primary tumors (UM-SCC-22A) but not from cells derived from the metastatic tumors (UM-SCC-22B).<br>● Blockade of VEGF from conditioned medium abrogated Akt phosphorylation in ALDH+/CD44+ cells from UM-SCC-22A (derived from primary tumor). There was a more modest effect on Akt phosphorylation in ALDH+/CD44+ cells from UM-SCC-22B or ALDH−/CD44− cells in UM-SCC-22A (derived from metastatic tumor)<br>● Exposure of control (ALDH−/CD44−) cells to the conditioned medium induced activation of Akt only in cells from the primary tumor (UM-SCC-22A) but not in cells from the metastatic tumor (UM-SCC-22B)

Experimental findings supporting the role of endothelial cell derived growth factors in regulating Epithelial Mesenchymal Transition and maintaining stemness of head and neck cancer stem cells.

**HNSCC = head and neck squamous cell carcinoma, *OSCC = oral squamous cell carcinoma , *SCC = squamous Cell Carcinoma, *SP = side population, *EMT = epithelial-mesenchymal transition, *EGF = Epidermal growth factor*
